# CD147 overexpression may serve as a promising diagnostic and prognostic marker for gastric cancer: evidence from original research and literature

**DOI:** 10.18632/oncotarget.15737

**Published:** 2017-02-25

**Authors:** Chenghao Hu, Xiaoxia Dong, Junbo Wu, Feifan Xiao, Jun Shang, Liang Liu, Yuan Yang, Dongmei Luo, Qiuting Li, Qian Song, Jingcheng Yang, Chengdong Zhang, Li Shen, Zhiguo Luo

**Affiliations:** ^1^ Department of Clinical Oncology, Taihe Hospital, Hubei University of Medicine, Shiyan, Hubei, PR China; ^2^ Department of Pharmacology, School of Basic Medicine, Hubei University of Medicine, Shiyan, Hubei, PR China; ^3^ Center for Evidence-Based Medicine and Clinical Research, Taihe Hospital, Hubei University of Medicine, Shiyan, Hubei, PR China; ^4^ First Clinical Academy, Guangxi Medical University, Nanning, Guangxi, PR China; ^5^ Department of Thoracic and Cardiovascular Surgery, The First Affiliated Hospital of Guangxi Medical University, Nanning, Guangxi, PR China; ^6^ Department of Radiation Oncology, Fudan University Shanghai Cancer Center, Fudan University, Shanghai, PR China; ^7^ School of Life Sciences, Fudan University, Shanghai, PR China; ^8^ School of Mathematics and Physics, Anhui University of Technology, Maanshan, Anhui, PR China; ^9^ Department of Clinical Oncology, Liyuan Hospital, Tongji Medical College, Huazhong University of Science and Technology, Wuhan, Hubei, PR China

**Keywords:** gastric cancer, CD147, original research, meta-analysis

## Abstract

Gastric cancer (GC) is one of the most common malignancies worldwide. The expression of CD147 protein is associated with GC. However, the clinical role of CD147 in GC has not been investigated extensively. Hence, we focused on studying the association between the expression of CD147 and clinicopathological features of GC patients in this study. Firstly, sixteen publications (1752 cases and 391 controls) and one from our own original research (143 cases) were included in the meta-analysis to obtain a more precise estimation of the diagnostic value of CD147. The results showed that expression rate of CD147 in the GC group is higher than that in control group. Moreover, gender, TNM stage, lymph node metastasis, and depth of invasion are all associated with CD147. Further, sections of gastric tissue from 143 cases underwent immunohistochemical staining for evaluation of CD147 protein expression. Our retrospective analysis demonstrated CD147 protein expression was significantly associated with clinical N stage, and tumor stage. Meanwhile, it can also serve as an independent prognosis biomarker. In conclusion, our results support the role of CD147 as a good indicator of diagnosis and prognosis.

## INTRODUCTION

Gastric cancer (GC) is one of the most common malignancies in the world and the second leading cause of global cancer death [1]. Based on the latest statistical study in the USA, 24,590 new cases of GC were diagnosed, and 10,720 deaths caused by this disease were recorded in 2015 [2]. A variety of risk factors are responsible for GC including smoking, drinking, male gender and infection with Helicobacter pylori [3]. Besides, genetic factor play an important role in the development of GC. Despite progress in multimodality therapy, the five-year survival rate remains low. Therefore, identification of factors that affect patient survival is critical for novel therapy development [4].

Extracellular matrix metalloproteinase inducer, also named as cluster of differentiation 147 (CD147) or basigin, is a widely distributed cell surface glycoprotein that is involved in numerous physiological and pathological functions, especially in tumor invasion and metastasis [5]. Riethdorf et al. [6] thought that CD147 increases angiogenesis via upregulation of VEGF and metalloproteinases, increased EGFR expression, and increased invasion and metastasis via MMP upregulation. Previously, Huang et al. [7] indicated that the expression of CD147 may be used as one of the objective marks to estimate the behaviors of GC. Gao et al.[8] also indicated that the overexpression of CD147 protein was positively correlated with GC.

In fact, meta-analysis can play a key role in generating new hypotheses [9]. In this research, a meta-analysis of all eligible studies was conducted to obtain a more precise estimation of the associations. Besides, we also hypothesized that CD147 has a prognostic value. Because the clinical role of CD147 expression in GC has not been extensively studied, a retrospective analysis of 143 cases was performed to explore the relationship between the expression level of CD147 protein and clinicopathological features (which also contribute to the meta-analysis) as well as clinical prognosis in GC patients.

## RESULTS

### Meta-analysis

#### Eligible studies

**Figure 1:**
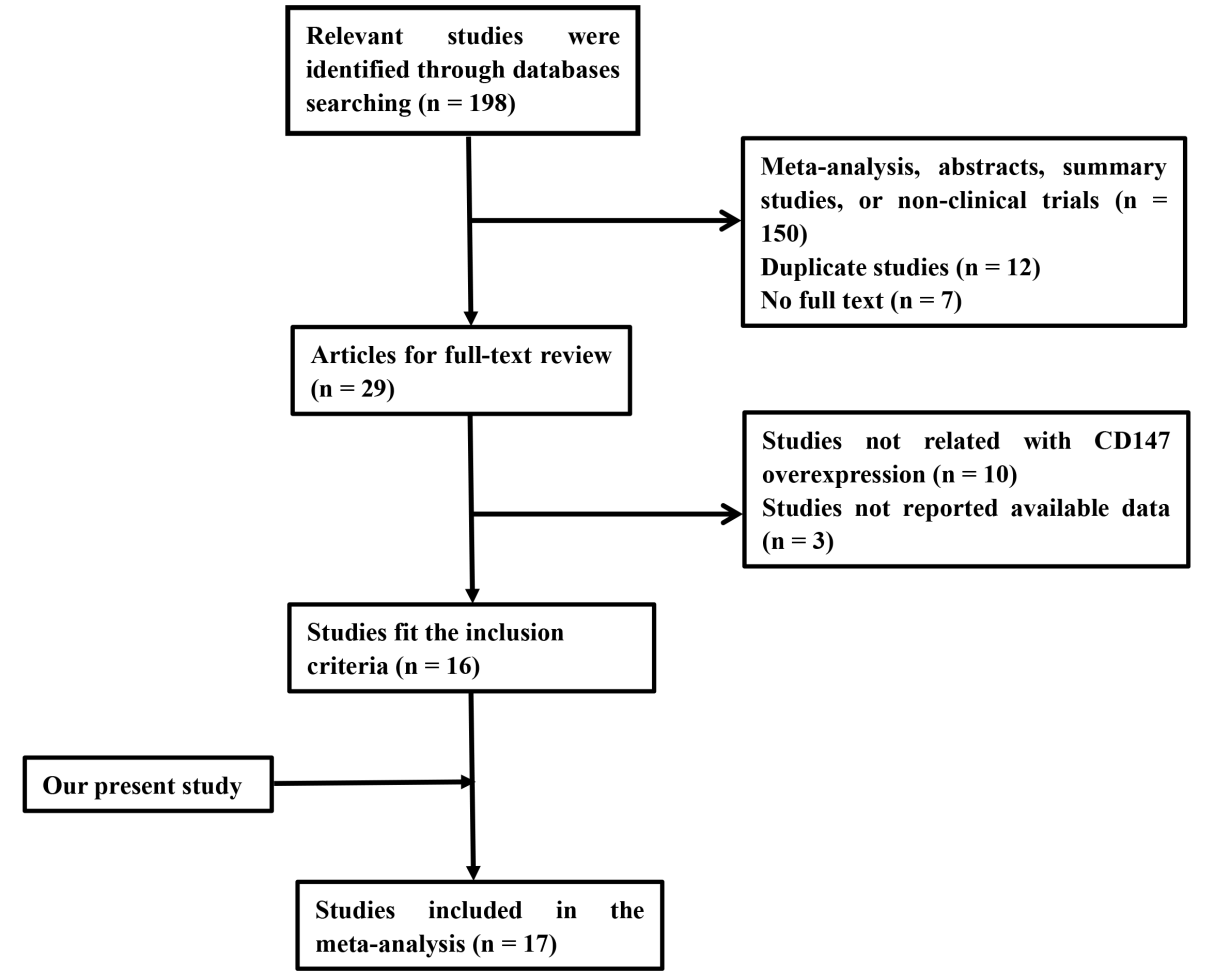
Flowchart showing the selection process for the included studies

Sixteen publications were included in our meta-analysis. Ten studies were included for overall analysis and other six studies were included for subgroup analysis. The screening process is presented in detail in Figure 1. The detail information were showed in the following analysis.

#### GC group *vs*. control group

**Figure 2:**
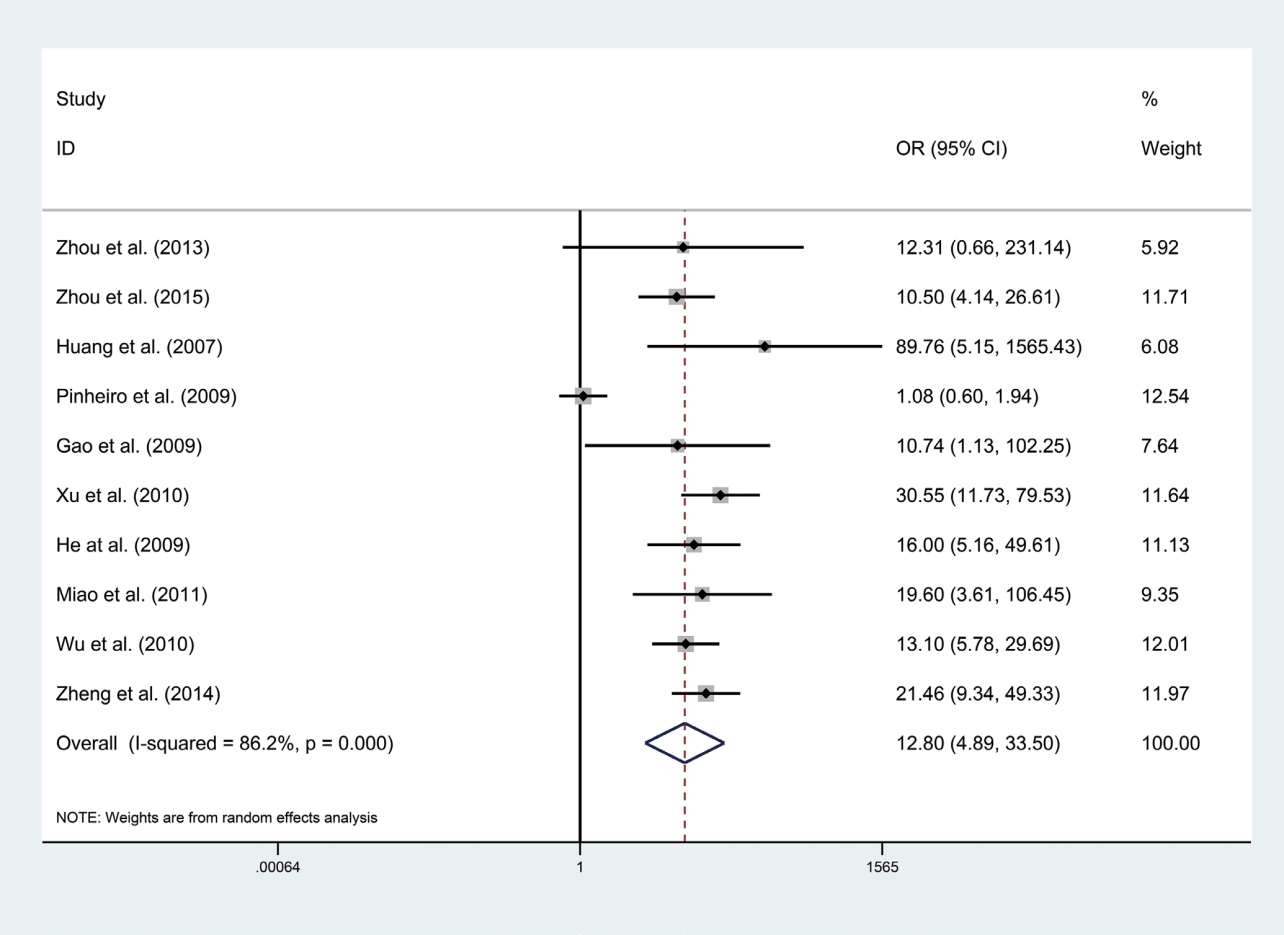
Forest plot of odd ratios (ORs) of 10 included studies using a random-effect model

**Figure 3:**
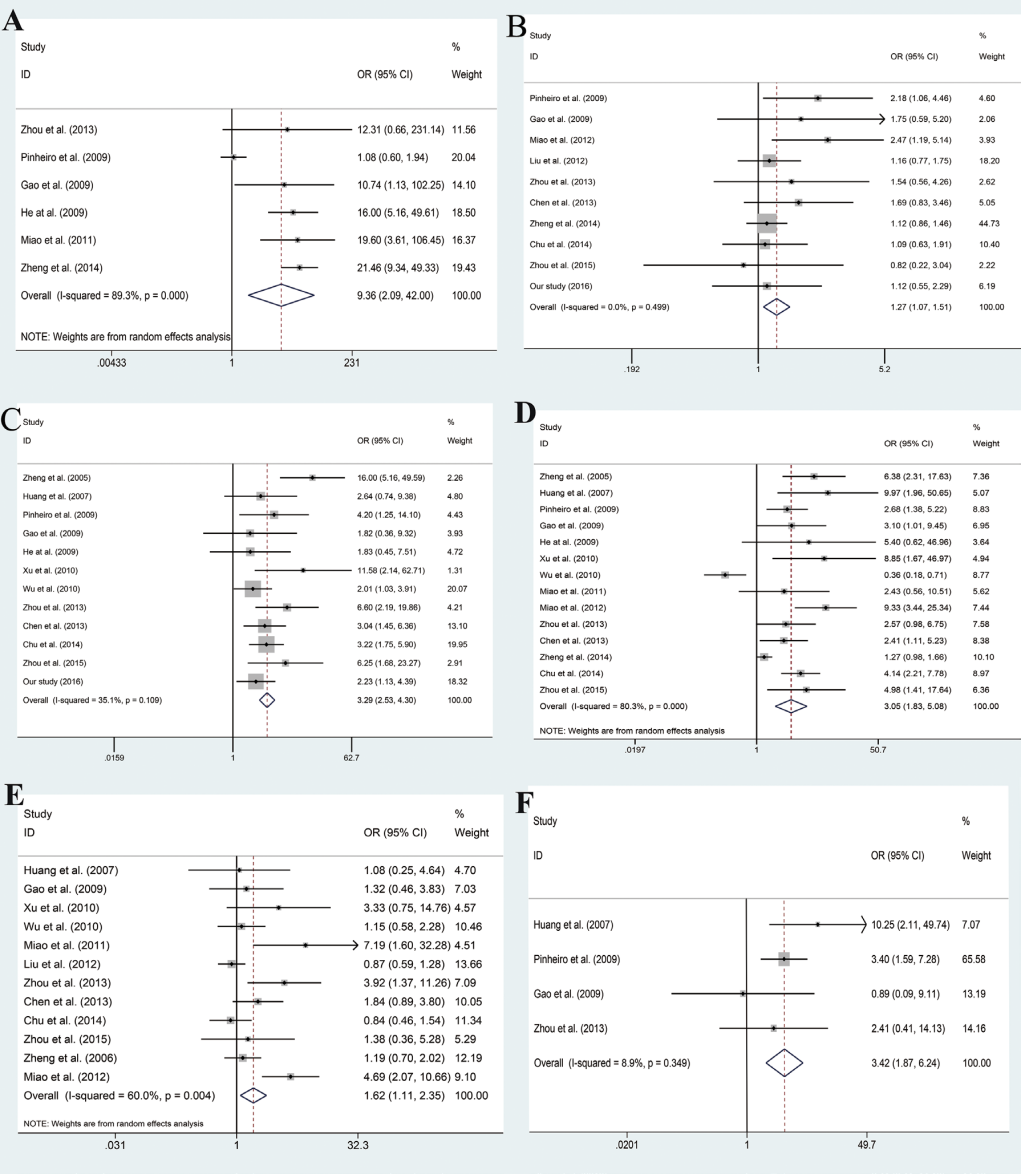
Forest plot of odd ratios (ORs) of subgroup analysis **A**. subgroup analysis based on control tissues. **B**. subgroup analysis based on gender. **C**. subgroup analysis based on TNM stage. **D**. subgroup analysis based on LN metastasis. **E**. subgroup analysis based on differentiation. **F**. subgroup analysis based on depth of invasion.

A total of 10 studies [7, 8, 10–17] (Table 1) which reported the expression of CD147 in GC group (GC tissues) and control group (pericarcinoma tissues or normal gastric tissues) were included for this analysis. Our results indicated that expression rate of CD147 in the GC group was higher than that in the control group, and the difference between the two groups was statistically significant (OR = 12.80, 95% CI: 4.89 - 33.50, *P* = 0.000, I^2^ = 86.2%, Figure 2). Furthermore, our meta-analysis demonstrated that CD147 expressions in GC tissues were significantly higher than normal mucosa tissues (OR = 9.36, 95% CI: 2.09 - 42.00, *P* = 0.000, I^2^ = 89.3%, Figure 3A).

**Table 1:** Characteristics of the case–control studies included in the meta-analyses

			Cases		Controls		
Author	Year^a^	Country	high expression	low expression	high expression	low expression	Tissue for cases
Zhou et al.	2013	China	37	33	0	5	Normal mucosa tissues
Zhou et al.	2015	China	49	14	10	30	Adjacent non-tumor tissues
Huang et al.	2007	China	40	18	0	20	Adjacent non-tumor tissues
Pinheiro et al.	2009	Portugal	66	94	26	40	Normal mucosa tissues
Gao et al.	2009	China	51	19	1	4	Normal mucosa tissues
Xu et al.	2010	China	56	9	11	54	Adjacent non-tumor tissues
He at al.	2009	China	40	10	6	24	Normal mucosa tissues
Miao et al.	2011	China	49	10	2	8	Normal mucosa tissues
Wu et al.	2010	China	131	30	10	30	Adjacent non-tumor tissues
Zheng et al.	2014	Japan	551	445	6	104	Normal mucosa tissues

^a^Year of publicate

#### Subgroup analysis by gender: male group *vs*. female group

A total of 10 studies [8, 10–12, 17–21] (including ours) (Supplementary Table S1) involving 2305 patients were included in this subgroup. Compared with female group, expression rate of CD147 is higher in male group. A significant statistical difference was observed (OR = 1.27, 95% CI: 1.07 - 1.51, *P* = 0.499, I^2^ = 0%, Figure 3B).

#### TNM stage of GC tissues: high stage group *vs*. low stage group

A total of 12 studies [7, 8, 10–14, 16, 18, 21, 22] (including ours) (Supplementary Table S2) reported the expression of CD147 in high stage group and low stage group of GC tissues. Meta-analysis of fixed effect model showed that expression rate of CD147 in the high stage group was higher than that in low stage group. The difference between the two groups was statistically significant (OR = 3.29, 95% CI: 2.53 - 4.30, *P* = 0.109, I^2^ = 35.1%, Figure 3C).

#### Lymph node (LN) metastasis of GC tissues: positive group *vs*. negative group

A total of 14 studies [7, 8, 10–19, 21, 22] (Supplementary Table S3) reported the expression of CD147 in positive and negative lymph node metastasis of GC tissues. Our result showed that expression rate of CD147 in the positive group (LN+) is higher than that in negative group (LN−). The difference between two groups was statistically significant (OR = 3.05, 95% CI: 1.83 - 5.08, *P* = 0.000, I^2^ = 80.3%, Figure 3D).

#### Differentiation of GC tissues: Poor group *vs*. Well and Moderate group

A total of 12 studies [7, 8, 10, 11, 13, 15, 16, 18–21, 23] (Supplementary Table S4) reported the expression of CD147 in well and moderate group and poor group of GC tissues. Our results showed that expression rate of CD147 in the poor group is higher than the Well and Moderate group. There was difference between two groups (OR = 1.62, 95% CI: 1.11 - 2.35, *P* = 0.004, I^2^ = 60.0%, Figure 3E).

#### Depth of invasion of GC tissues: Muscular propria/subserosa *vs*. Mucosa

A total of 4 studies [7, 8, 10, 12] (Supplementary Table S5) reported the expression of CD147 in Muscular propria/subserosa group and Mucosa group of GC tissues. Our result showed that expression rate of CD147 in the Muscular propria/subserosa group is higher than that in Mucosa group. The difference between two groups was statistically significant (OR = 3.42, 95% CI: 1.87 - 6.24, *P* = 0.349, I^2^ = 8.9%, Figure 3F).

#### Publication bias and sensitivity Analysis

The publication bias was assessed by Begg's funnel plot and Egger's test. The results showed there were no publication bias in the analysis (Data not shown). The stability of the study was detected by sensitivity analysis, by excluding one individual study in sequence. Our analysis displayed that result was not changed.

### CD147 expression is closely associated with clinicopathological features in GC patients

**Figure 4:**
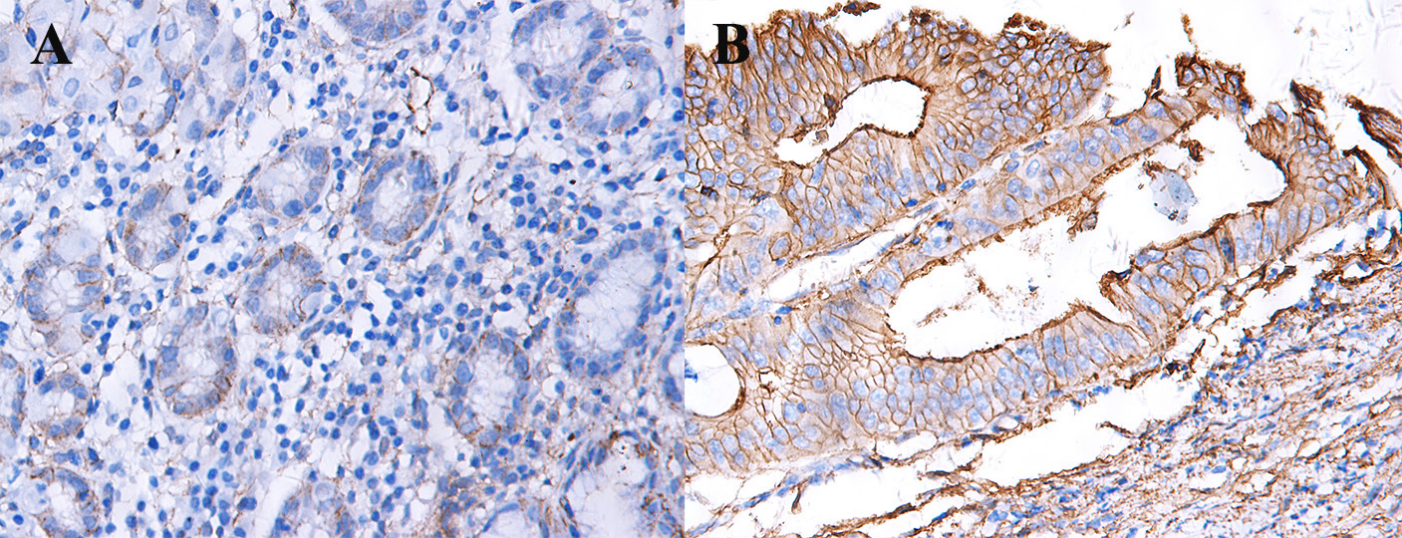
Expression of CD147 in GC and adjacent non-tumor tissues **A**. expression of CD147 in GC tissues. **B**. expression of CD147 in adjacent non-tumor tissues.

We calculated the expression levels of CD147 protein in 143 paraffin-embedded GC samples using immunohistochemical staining. The power analysis indicates our sample size is enough (Power = 0.9997). As shown in Figure 4, CD147 expression is more frequent in GC tissues than that in adjacent non-tumor tissues. The relationship between the expression level of CD147 protein and the clinicopathological parameters of GC was analyzed. As summarized in Table 2, overexpression of CD147 protein is significantly associated with clinical N stage (N0 vs. N1-N3; *P* = 0.011), and tumor stage (stage I-II vs. stage III-IV; *P* = 0.019).

**Table 2:** Relationship between CD147 expression and clinicopathological features of gastric cancer patients

Clinical parameter	No. of patients	CD147		*P*-value
		low(*n*=67)	high(*n*=76)	
Age				
≤65	73	36	37	0.547
>65	70	31	39	
Gender				
male	100	46	54	0.755
female	43	21	22	
tumor_max_diameter				
d<5	73	37	36	0.348
d≥5	70	30	40	
T				
T1-2	29	15	14	0.556
T3-4	114	52	62	
N				
N0	37	24	13	0.011
N1-3	106	43	63	
M				
M0	136	65	71	0.448
M1	7	2	5	
stage				
ࡰ+Ⅱ	60	35	25	0.019
Ⅲ+Ⅳ	83	32	51	

### Correlation between CD147 expression and clinical outcome in subtypes of GC

**Figure 5:**
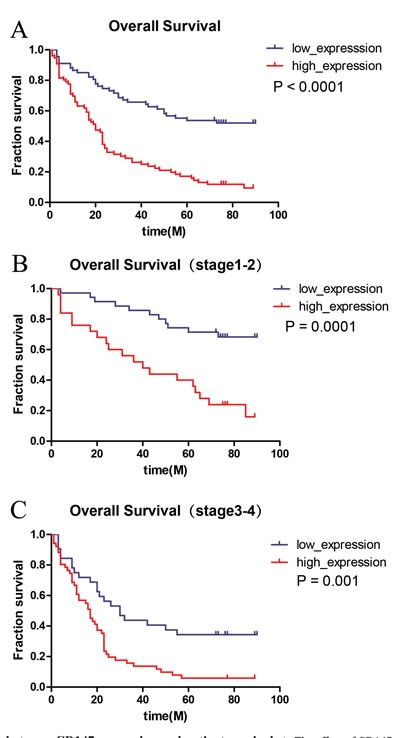
Association between CD147 expression and patient survival **A**. The effect of CD147 expression on OS on the whole (log-rank *p* < 0.05). **B**., **C**. OS were significantly different between the low expression group and the high expression group both in low stage(stage I-II) and high stage(III-IV) (log-rank *p* < 0.05).

In order to investigate the association between CD147 expression and clinical outcome, we divided the patients into CD147-high and CD147-low groups, and survival analyses were performed using the Kaplan-Meier method. In summary, the patients with higher CD147 expression had significant lower OS (P < 0.0001) than those with lower CD147 expression (Figure 5A). Meanwhile, the results of survival analyses coming from the low stage (stage I-II) groups (*P* = 0.0001, Figure 5B), and the high stage (stage III- IV) groups (*P* = 0.001, Figure 5C) demonstrated that the OS was lower in patients with higher expression of CD147.

### Univariate and Multivariate analyses of OS in patients with GC

**Figure 6:**
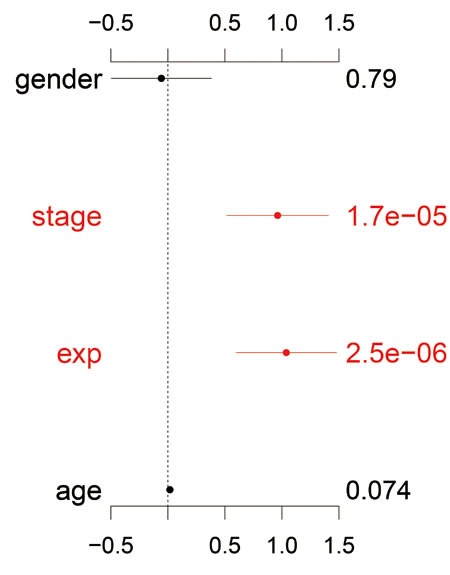
Multivariate analysis of overall survival CD147 expression as an independent prognostic factor in gastric cancer.

Based on the result of univariate analysis, it demonstrated that CD147 overexpression was significantly associated with poor overall survival (OS) (HR = 3.206, 95% CI = 2.092-4.915, *P* = 8.95E-08). In multivariate survival analysis, T, N, and M collapsing into stage as a whole, together with age and gender, were included in the Cox proportional hazard models as adjusted variables. Significantly, overexpression of CD147 is negatively correlated with OS (HR = 2.825, 95% CI = 1.833-4.352, *P* = 2.51E-06), which indicated that CD147 can act as an independent predict factor for GC patients (Table 3). The other meaningful prognostic factors for OS in GC is tumor stage (HR = 2.618, 95%CI = 1.689-4.056, *P* = 1.66E-05) which has been universally acknowledged (Figure 6).

**Table 3:** Univariateand multivariate analysis of overall survival in 90 patients with gastric cancer

Factor	Univariate analysis		Multivariate analysis	
	HR(95%CI)	*P*	HR(95%CI)	*P*
Age	1.021(1.001-1.041)	0.039	1.018(0.998-1.038)	0.074
Gender	0.928( 0.605-1.424)	0.733	0.944(0.614-1.453)	0.794
Stage	2.898(1.883- 4.461)	1.32E-06	2.618(1.689-4.056)	1.66E-05
Expression	3.206(2.092-4.915)	8.95E-08	2.825(1.833-4.352)	2.51E-06

## DISCUSSION

To the best of our knowledge, this is the first meta-analysis, which included sixteen published studies (1752 cases and 391 controls) and one original study (143 cases) from our own research, to assess the association between CD147 and the clinicopathological features of GC. Our results indicate that expression rate of CD147 in the GC group is higher than that in control group. Besides, gender, TNM stage, lymph node metastasis, and depth of invasion have a relationship with the expression state of CD147. Further, our retrospective analysis of 143 cases demonstrated CD147 protein expression can be an independent prognosis biomarker for GC with a strong statistical power.

In recent years, although surgery, chemoradiotherapy, and targeted therapy make great progress to prolong survival of GC patients, GC is still the third leading cause of cancer-related death worldwide mainly due to tumor local recurrence and distant metastasis [24]. With the development of related genomic and molecular technology, some biomarkers for GC diagnosis and treatment were discovered. However, sensitive biomarkers which are able to diagnose GC in early stage and predict prognosis of GC patients are needed.

CD147 is a 57-kilodalton (kDa) transmembrane glycoprotein which belongs to the immunoglobulin (IgG) superfamily [25]. Moreover, CD147, comprised of 2 extracellular Ig domains, is a single transmembrane domain required for counter receptor binding activity which is involved in matrix metalloproteinases (MMPs) induction and a short cytoplasmic domain that interacts with Cav 1 [26, 27]. CD147 mediates tumor cell-macrophage interactions, and has been shown to induce both MMPs and vascular endothelial growth factor (VEGF) [28]. VEGF plays an important role in angiogenesis [29]. It is notable that angiogenesis is necessary for tumor growth and metastasis and constitutes an important point in the control of cancer progression [30]. Frequently, tumors overexpress proangiogenic factors, such as vascular endothelial growth factor, allowing them to make this angiogenic switch [31].

Based on our meta-analysis, results showed that the expression rate of CD147 in the GC group was higher than that in control group. Furthermore, CD147 expressions in GC tissues were significantly higher than normal mucosa tissues. When exploring the diagnostic value of CD147 in GC by immunohistochemistry, we found that CD147 expression level was higher in GC tissues than in adjacent non-tumor tissues. Similar with our study, Xu et al. [13] and Wu et al. [16] found that CD147 expression in GC tissues was higher than that in paired adjacent non-tumor tissues. Except for GC, CD147 was found relevant in other malignancies like colorectal cancer [32], ovarian cancer [33], and clear cell renal cell carcinoma [34]. Thus, it is not surprising that the expression rate of CD147 is higher in GC tissues.

Previous studies have identified gender differences that may contribute to the development of GC [35, 36]. It may reflect that the role of biological factors is important and suggest that female might be intrinsically more vigorous than men in coping with cancer [37]. However, our research showed there was no significant statistic between male group and female group. Interestingly, the expression rate of CD147 in male group was higher than that in female group in our meta-analysis. Due to the lack of detailed source data, we cannot perform a subgroup analysis based on age. Thus, more research should be done in the future.

Invading basement membrane (BM) barriers is essential steps in the pathology of GC [38]. Depth of invasion is the most important predictors of survival [39]. The deeper the invasion into the stomach wall, the poorer the prognosis will be [40]. So, the meta-analysis showed that expression rate of CD147 in the Muscular propria/subserosa group is higher than that in Mucosa group.

The TNM Classification of Malignant Tumors (TNM) is a cancer staging notation system that gives codes to describe the stage of a person's cancer. The higher stages represent higher progression of cancer. Previously, Wu et al. [16] indicated that CD147 was over expressed in high stage compared with low stage. Our meta-analysis also showed that expression rate of CD147 in the high stage group was higher than that in low stage group. In fact, due to the higher malignancy, it is not surprising that the high stage tissues express more CD147 than the low stage tissues. In the analysis of the association between CD147 and clinicopathological features of GC patients, our retrospective research proved that TNM stage and N stage were associated with expression of CD147. In addition, Miao et al. [19] demonstrated that overexpression of CD147 is associated with Lymph node (LN) metastasis of GC. Based on our meta-analysis, our result confirmed that expression rate of CD147 in the positive group (LN+) is higher than that in negative group (LN−). In fact, lymph node metastasis is the most common way for tumor metastasis. Even in early GC (EGC), the incidence of LN metastasis exceeds 10% [41]. GC with lymph node metastasis has more damage to patient which means more serious patient's condition.

After analyzing the cases with clinic information in our retrospective data, we found that the OS of the GC group with low CD147 expression had a longer survival time than that of high CD147 expression group. The univariate and multivariate analyses also demonstrated that CD147 overexpression was significantly associated with poor OS. Notably, the patients with lower CD147 expression showed a longer survival time compared with high CD147 expression patients in low stage group and high stage group. This may indicate that the level of CD147 expression can be used as a molecular biomarker for the prognosis of GC. Since good prognostic prediction is necessary to ascertain the risk and the effectiveness of treatments such as surgery and chemoradiotherapy, CD147 may be a competent prognostic marker to predict treatment response.

However, several limitations about our research should be addressed. First, there are high heterogeneity in GC group vs. control group in the meta-analysis. After we excluded Pinheiro et al.'s study [12], I squared turn to 0, but the result did not changed. A possible reason is that population of this study were Portugal, which different with other studies. Second, except 3 studies published in English, the other 13 studies were all in Chinese, the language bias is unavoidable. Third, most study participants were Chinese, so the results may not be generalizable to other races. Fourth, due to the limitation of included studies, we cannot assess the role of CD147 in prediction the response of treatment. Thus, further studies with larger numbers of patients and more comprehensive clinical data are warranted to explore the full picture of the role of CD147 in GC.

Above all, CD147 upregulated in GC tissues compared with noncancerous tissues. Moreover, gender, TNM stage, lymph node metastasis, and depth of invasion are all associated with CD147. Our retrospective analysis demonstrated CD147 protein expression was significantly associated with clinical N stage, and tumor stage. Meanwhile, it can also serve as an independent prognosis biomarker. In conclusion, our results support the role of CD147 as a good indicator of diagnosis and prognosis.

## MATERIALS AND METHODS

### Meta-analysis

#### Literature search

Eligible publications were retrieved by searching PubMed, Embase, and Chinese National Knowledge Infrastructure (CNKI) databases up to April 22, 2016. The search strategy was based on the following words: (“antigens, cd147”[MeSH Terms] OR (“antigens”[All Fields] AND “cd147”[All Fields]) OR “cd147 antigens”[All Fields] OR “cd147”[All Fields]) AND (“stomach neoplasms”[MeSH Terms] OR (“stomach”[All Fields] AND “neoplasms”[All Fields]) OR “stomach neoplasms”[All Fields] OR (“gastric”[All Fields] AND “cancer”[All Fields]) OR “gastric cancer”[All Fields]). Furthermore, we also searched the additional publications from the reference lists of the retrieved articles or reviews which had been previously missed.

#### Inclusion and exclusion criteria

In this meta-analysis, publications that met the following criteria were selected as candidate articles: (1) inclusion of pathologically confirmed GC patients; (2) investigation of the relationship between the expression of CD147 and GC; (3) available data for calculation of odds ratios (ORs) with their corresponding 95% confidence intervals (CIs). And the following exclusion criteria were used: (1) abstracts and reviews, (2) insufficient data to extract or calculate the ORs, and (3) repeated or overlapping publications.

#### Data extraction

Two authors identified and screened the search findings. A third author reviewed all data entries. The following items were extracted from the eligible studies: name of the first author, publication year, country of the first author, sample size and numbers of cases and controls.

#### Statistical analysis

In this meta-analysis, all statistical analyses were performed using the STATA 12.0 software (StatCorp, College Station, TX, USA). Pooled odds ratios (ORs) and 95% confidence intervals (CIs) were calculated to evaluate the strength of the associations. We judged heterogeneity by calculating the I^2^ statistic, where an I^2^ value from 0 to 25% indicate low heterogeneity, 25-50% moderate heterogeneity and ≥ 50% high heterogeneity. If data was not significantly heterogeneous (P > 0.05 or I^2^ < 50 %), the pooled effects were calculated using a fixed model, Otherwise, a random-effects model was employed [42]. Publication bias was evaluated using the Egger's and Begg's test. We also employed sensitivity analysis to evaluate stability of the results.

### Study population

Patients with GC were recruited from a study carried out between 2009 and 2014 at Taihe Hospital, Hubei University of Medicine. Clinic pathologic data for parameters were collected from the pathology report. None of the patients had received radiotherapy or chemotherapy before surgery. From their medical records the data on date of diagnosis, age, sex, tumor size, lymph node metastasis, TNM stage (TNM stages I and II were classified as low stage, III and IV as high stage), and the overall survival were extracted. The study has been approved by the institutional review board at Hubei University of Medicine. All methods used in this study were carried out in accordance with the approved guidelines and all experimental protocols were approved by Hubei University of Medicine. Informed consent was obtained from all subjects.

### Immunohistochemistry

After surgical procedures, clinical tissue samples were fixed in 4% paraformaldehyde. Before cutting into 4 μm sections, the samples were embedded in paraffin. The sections were dewaxed and rinsed in 100% xylene for 10 minutes. The sections were then rehydrated in 100% alcohol, 95% alcohol, 90% alcohol, 80% alcohol and 70% alcohol for 5 minutes. Then, 5 % hydrogen peroxide was applied to block endogenous peroxide activity and the samples were washed with phosphate-buffered saline (PBS). Blocking was performed with 5% sodium citrate solution for 15 min at 100 °C. The sections were incubated at 4°C overnight with the anti-CD147 antibody (1:500) (Sigma, St. Louis, MO, USA). Next day, the slides were washed with PBS and incubated with secondary antibody for 20 minutes at 35 °C, followed by color development with Diaminobenzidine (DAB) for 3 minutes after washed with PBS. The cell nuclei were counterstained with hematoxylin for 3 minutes. Sections were photographed on an Olympus photomicroscope (Inha, Japan). The degree of IHC staining was evaluated by two independent pathologists. Staining intensity was graded as “0” (negative), “1” (weak), “2” (moderate) and “3” (strong); staining percentage was graded as “0” ( < 5%), “1” (5-25%), “2” (25-50%), “3” (50-75%) or “4” (>75%). A final immunoreactivity scores (IRS) was calculated by multiplying the values of the staining intensity and staining percentage. The IRS value > 4 was defined as high expression and IRS value ≤ 4 as low expression. ImagePro Plus (Media Cybernetics, Silver Spring, MD, USA) was used to quantitatively score the tissue sections.

### Statistic method

The SPSS 19.0 (IBM Corporation, Armonk, NY, USA), GraphPad Prism Software 5 (GraphPad, Inc.; La Jolla; California; USA) and R software (R3.3.0) for windows was used for statistical analysis. Correlation between CD147 expression and clinicopathological characteristics were evaluated by Chi-square test and Fisher's exact tests. Overall survival (OS) rate were estimated by using the Kaplan-Meier method and the log-rank test was used to calculate P Values. Univariate and multivariate Cox regression models were used to calculate hazard ratios and their confidence intervals for the study variables. A one-sided power calculation was performed on the effect size (hazard ratio) of CD147 expression in Cox regression [43]. Historically, the value of 0.80 (Beta = 0.20) was used for power. All the other analyses were considered statistically significant when a two-sided P < 0.05.

## SUPPLEMENTARY MATERIALS TABLES



## Abbreviations

(GC): Gastric cancer
(CD147): cluster of differentiation 147
(kDa): kilodalton
(IgG): immunoglobulin
(BM): basement membrane
(PBS): phosphate-buffered saline
(DAB): Diaminobenzidine
(ORs): odds ratios
(CIs): confidence intervals
(EGC): early GC
