# Supplementary material for: CD147 overexpression may serve as a promising diagnostic and prognostic marker for gastric cancer: evidence from original research and literature
Source: Oncotarget. 2017 Feb 25;8(19):30888–99. doi: 10.18632/oncotarget.15737 (PMC5458175; doi:10.18632/oncotarget.15737)
Supplement: Supplementary file 1 [file oncotarget-08-30888-s001.docx]

Title: CD147 overexpression may serve as a promising diagnostic and prognostic marker for gastric

cancer: evidence from original research and literature

Author list: Hu Chenghao, Dong Xiaoxia, Wu Junbo, Xiao Feifan, Zhang Chengdong, Yang Jingcheng, Yang Yuan,

Liu Liang, Shang Jun, Luo Dongmei, Li Qiuting, Song Qian, Shen Li, Luo Zhiguo

Supplementary Table S1: Characteristics of the case–control studies included for subgroup analysis based on gender

|  |  | male | | female | |
| --- | --- | --- | --- | --- | --- |
| First author | Year | high expression | low expression | high expression | low expression |
| Pinheiro et al. | 2009 | 49 | 54 | 15 | 36 |
| Gao et al. | 2009 | 36 | 11 | 15 | 8 |
| Miao et al. | 2012 | 90 | 16 | 50 | 22 |
| Liu et al. | 2012 | 193 | 108 | 85 | 55 |
| Zhou et al. | 2013 | 27 | 21 | 10 | 12 |
| Chen et al. | 2013 | 44 | 26 | 28 | 28 |
| Zheng et al. | 2014 | 250 | 386 | 132 | 228 |
| Chu et al. | 2014 | 42 | 78 | 34 | 69 |
| Zhou et al. | 2015 | 33 | 10 | 16 | 4 |
| Our study | 2016 | 54 | 46 | 22 | 21 |

Supplementary Table S2: Characteristics of the case–control studies included for subgroup analysis based on TNM stage.

|  |  | high stage | | low stage | |
| --- | --- | --- | --- | --- | --- |
| First author | Year | high expression | low expression | high expression | low expression |
| Zheng et al. | 2005 | 85 | 5 | 17 | 16 |
| Huang et al. | 2007 | 28 | 5 | 17 | 8 |
| Pinheiro et al. | 2009 | 10 | 4 | 50 | 84 |
| Gao et al. | 2009 | 9 | 2 | 42 | 17 |
| He at al. | 2009 | 22 | 4 | 18 | 6 |
| Xu et al. | 2010 | 43 | 2 | 13 | 7 |
| Wu et al. | 2010 | 60 | 21 | 47 | 33 |
| Zhou et al. | 2013 | 22 | 6 | 15 | 27 |
| Chen et al. | 2013 | 51 | 24 | 21 | 30 |
| Chu et al. | 2014 | 56 | 67 | 20 | 77 |
| Zhou et al. | 2015 | 35 | 4 | 14 | 10 |
| Our study | 2016 | 51 | 32 | 25 | 35 |

Supplementary Table S3: Characteristics of the case–control studies included for subgroup analysis based on LN metastasis

|  |  | positive | | negative | |
| --- | --- | --- | --- | --- | --- |
| First author | Year | high expression | low expression | high expression | low expression |
| Zheng et al. | 2005 | 87 | 10 | 15 | 11 |
| Huang et al. | 2007 | 29 | 2 | 16 | 11 |
| Pinheiro et al. | 2009 | 42 | 37 | 22 | 52 |
| Gao et al. | 2009 | 30 | 6 | 21 | 13 |
| He at al. | 2009 | 15 | 1 | 25 | 9 |
| Xu et al. | 2010 | 43 | 2 | 17 | 7 |
| Wu et al. | 2010 | 30 | 28 | 77 | 26 |
| Miao et al. | 2011 | 25 | 3 | 24 | 7 |
| Miao et al. | 2012 | 82 | 5 | 58 | 33 |
| Zhou et al. | 2013 | 22 | 12 | 15 | 21 |
| Chen et al. | 2013 | 56 | 32 | 16 | 22 |
| Zheng et al. | 2014 | 162 | 227 | 214 | 382 |
| Chu et al. | 2014 | 59 | 67 | 17 | 80 |
| Zhou et al. | 2015 | 36 | 5 | 13 | 9 |

Supplementary Table S4: Characteristics of the case–control studies included for subgroup analysis based on differentiation.

|  |  | Poor | | Well and Moderate | |
| --- | --- | --- | --- | --- | --- |
| First author | Year | high expression | low expression | high expression | low expression |
| Huang et al. | 2007 | 11 | 3 | 34 | 10 |
| Gao et al. | 2009 | 25 | 8 | 26 | 11 |
| Xu et al. | 2010 | 35 | 3 | 21 | 6 |
| Wu et al. | 2010 | 60 | 28 | 41 | 22 |
| Miao et al. | 2011 | 37 | 3 | 12 | 7 |
| Liu et al. | 2012 | 154 | 96 | 124 | 67 |
| Zhou et al. | 2013 | 19 | 7 | 18 | 26 |
| Chen et al. | 2013 | 36 | 19 | 36 | 35 |
| Chu et al. | 2014 | 22 | 48 | 54 | 99 |
| Zhou et al. | 2015 | 38 | 10 | 11 | 4 |
| Zheng et al. | 2006 | 43 | 51 | 58 | 82 |
| Miao et al. | 2012 | 83 | 9 | 57 | 29 |

Supplementary Table S5: Characteristics of the case–control studies included for subgroup analysis based on depth of invasion.

|  |  | T2+T3 | | T1 | |
| --- | --- | --- | --- | --- | --- |
| First author | Year | high expression | low expression | high expression | low expression |
| Huang et al. | 2007 | 41 | 8 | 3 | 6 |
| Pinheiro et al. | 2009 | 48 | 47 | 12 | 40 |
| Gao et al. | 2009 | 48 | 18 | 3 | 1 |
| Zhou et al. | 2013 | 35 | 29 | 2 | 4 |
